# Thoracoscopy as a safe and effective technique for exploring calves affected with bovine respiratory disease

**DOI:** 10.1186/s40781-017-0129-5

**Published:** 2017-03-01

**Authors:** Natividad Perez-Villalobos, Iñaki Espinosa-Crespo, José Sampayo-Cabrera, Juan-Vicente González-Martín, Antonio Gonzalez-Bulnes, Susana Astiz

**Affiliations:** 1 TRIALVET S.L. C/Encina 22, 28721 Madrid, Spain; 2 Veterinary Practitioner: C/Foresta 42, 28760 Madrid, Spain; 3 VES S.L. C/Ferraz 31, 28008 Madrid, Spain; 4 Facultad Veterinaria (UCM), Avda Pta de Hierro s/n, 28040 Madrid, Spain; 5 Departamento Reproducción Animal (INIA), Avda Pta de Hierro s/n, 28040 Madrid, Spain

**Keywords:** Thoracoscopy, Bovine Respiratory Disease, Calves, Suitability, Field conditions

## Abstract

**Background:**

Bovine respiratory disease (BRD) is one of the leading causes of economic losses in the beef and dairy industry. Reliable antemortem tools for diagnosing BRD would improve the efficacy of treatment and reduce costs. Here we examined whether the relatively simple technique of thoracoscopy can support BRD diagnosis under field conditions. We also compared various equipment set-ups in order to optimize the safety and efficacy of the procedure. A total of 24 thoracoscopic procedures were performed in 17 calves diagnosed with BRD and in 2 healthy control calves. Rigid and flexible endoscopes and industrial videoscopes were tested using various insertion approaches. The suitability of the technique was assessed in terms of duration, volume of air extracted, visualization score, and image quality. Safety was assessed in terms of rectal temperature, body weight, breaths/min, presence of fibrinogen, pain score, recovery time, intraoperative complications and risk of laceration or threatening collapse.

**Results:**

Insertion of a flexible endoscope via a right, dorso-caudal approach at the 5^th^ intercostal space allowed complete examination of the right lung in 15 min, as well as identification of main lung lesions and adherences in calves with BRD, without compromising calf welfare. While the dorso-caudal approach was optimal, it was associated with substantial discomfort when rigid endoscopes were used, minimal complications or mortality due to thoracoscopy were observed up to 28 days after the procedure. Videoscopes were as safe and easy to use as endoscopes, but endoscopes provided better image quality.

**Conclusion:**

This study provides the first field evidence that thoracoscopy can be safe to explore BRD-diseased calves. These results justify a larger study to rigorously assess the diagnostic performance of the technique.

## Background

Bovine respiratory disease (BRD) is a major health issue in calves [1–7]. It is associated with greater rearing costs, higher risk of mortality and relapse, lower growth and early culling [8]. Post-mortem examination of lung lesions is a useful indicator of BRD prevalence [9], for which it shows specificity and sensitivity >75% [10]. Studies using this technique suggest that up to 17% of slaughtered calves in the Netherlands show extensive lung lesions [11]. However, post-mortem examination does not aid in the identification or treatment of currently affected animals [9], highlighting the need for reliable ante-mortem diagnostic tools that may improve both prognosis and treatment outcomes [12].

Ultrasonographic diagnosis of BRD in living animals is based on the sign of ‘lung consolidation’ [13–17]. This sign is routinely used to assess subclinical BRD [15], and it remains the best tool available for analysis of living animals [14, 16], particularly in feedlots [18]. However, this technique has limitations [19–21]. Researchers have raised concerns that diagnosis based on it may not always be reliable because internationally standardized ultrasound criteria for BRD are lacking [22]. In addition, ultrasound may be less reliable in the specific case of older feedlot calves, in which the presence and position of the squeeze chute and forelimb muscles can prevent clear imaging of the cranial part of the lung, which is the first lung tissue to be affected in BRD [23].

A complementary technique to ultrasound may be thoracoscopy, a minimally invasive, low-risk technique [24, 25] that allows assessment of intrathoracic processes. While images obtained by thoracoscopy can complement and support ultrasound images, the technique simultaneously allows the collection of tissue samples (biopsies), which have proven to be effective for diagnosing disease in other animal species [26–28], such as equines [24, 27, 29–31]. Thoracoscopy shows potential in cattle, based on two reports in healthy adult cows [25, 32] and one report in an adult cow with pericardiectomy [33]. It remains unclear whether thoracoscopy is effective on a larger scale and under field conditions.

The aim of the present study was to determine whether thoracoscopy in calves with BRD is safe and effective under field conditions. We also hoped to determine the optimal equipment and procedure to guide future research and implementation of this innovative method.

## Methods

This study involved 10 Holstein and 9 cross-breed calves from commercial feedlots (body weight, 132.39 ± 76.82 kg), of which 17 were suffering from BRD based on the following criteria: previous episodes with body temperature ≥40 °C, at least two clinical signs of BRD (cough, purulent nasal discharge, dyspnea and/or polypnea), at least two previous unsuccessful antibiotic treatments and growth retardation or weight loss [34, 35]. The two calves without BRD served as healthy controls in the study to assess the safety of the technique. During Phase 2 of the study (see Study design below), one control calf was euthanized the day after thoracoscopy with the farmer’s consent because of reduced expected performance, due to a previous femur fracture. Another calf was euthanized 7 days after thoracoscopy when persistently BVD infection was confirmed.

Procedures were carried out at the Large Animal Hospital at the Veterinary Faculty of Complutense University (Madrid, Spain) or at the animals’ farms, as indicated in Methods. Procedures complied with the Spanish Policy for Animal Protection (RD 1201/05), which fulfills European Union Directive 86/609 on the protection of animals.

### Endoscopes and industrial videoscopes

**Fig. 1:**
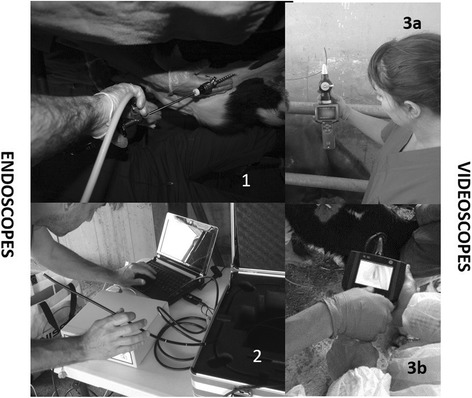
Evaluation of different endoscope and videoscope equipment set-ups for performing thoracoscopies in calves chronically affected with BRD. Set-up 1 involves a rigid endoscope; set-up 2, a flexible endoscope; set-up 3a, an industrial videoscope with low-quality imaging capability; and set-up 3b, an industrial videoscope with high-quality imaging capability

Four different equipment set-ups were used (Fig. 1):

- Set-up 1: rigid endoscope (Storz, Tuttlingen, Germany) with a length of 1 m and diameter of 4 mm, and laparoscopy cannula (Terman-type, Germany) with an internal diameter of 5 mm.
- Set-up 2: flexible bronchoscope (Storz), with a working channel, a diameter of 6 mm and a length of 1.5 m.
- Set-up 3a: portable industrial videoscope (Panter F3000, Madrid, Spain) with low-quality imaging capability, 170° rotation capability, length of 1 m and diameter of 5 mm, and other characteristics similar to those of the flexible endoscope.
- Set-up 3b: portable industrial videoscope (XL Vu Sensing & Inspection, GE, Naples, Italy) with high-quality imaging capability, 80° rotation capability, length of 1 m and diameter of 5 mm.

The only adjustment required for visualization was manually optimizing the light intensity depending on the distance between the end of the endoscope and the lung tissue. In practice, this intensity was similar across all the set-ups.

### Study design

#### Phase 1 (pilot testing of procedures on three calves in hospital)

**Fig. 2:**
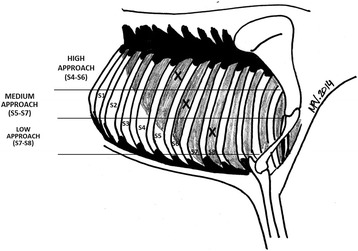
Evaluation of different approach areas for inserting the thoracoscope into calves chronically affected with BRD. X = recommended approach in the respective area; S = intercostal space in the recommended area. Intercostal spaces are shown in inverted order (from caudal to cranial) to facilitate interpretation by endoscopists

Three Holstein calves with medical history of chronic BRD and clinical BRD at the time of the study, were hospitalized and monitored daily. A rigid endoscope was used to perform thoracoscopy once in all animals (set-up 1, Fig. 1). At 22 daysays after the first procedure, a second thoracoscopy was performed in all animals to determine whether the first thoracoscopy had caused any lesions. Two different approaches out of the three possible ones (dorso-caudal, medio-cranio-ventral or cranio-ventral; Fig. 2) were performed in each thoracoscopy, in the same calf. Therefore, Phase 1 involved 6 thoracoscopies and 8 approaches. Results were analyzed based only on the type of approach (dorso-caudal, medio-cranio-ventral or cranio-ventral), since the animals were assumed to be similar in their clinical and other characteristics.

#### Phase 2 (18 procedures in 16 calves; 15 in feedlots and 3 in hospital)

As a result of our experiences during Phase 1, we decided to use only flexible equipment (set-ups 2, 3a and 3b; Fig. 1) in the dorsal approach (dorso-caudal at 5^th^ intercostal space, Fig. 2). A total of 18 thoracoscopies were performed in 16 calves (7 Holstein and 9 crossbreed): 5 with a flexible endoscope (set-up 2), 9 with an industrial videoscope offering low image quality (set-up 3a) and 4 with an industrial videoscope offering high image quality (set-up 3b). All calves had a medical history of chronic BRD and clinical BRD at the time of thoracoscopy, except for two healthy calves that served as controls to assess the safety of the technique with these particular endoscopes. Three thoracoscopic procedures (one each with set-up 2, 3a or 3b) were performed under experimental conditions in hospitalized calves, while the other 15 thoracoscopies were performed under field conditions. Results were analyzed on the basis of set-up (1, 2, 3a or 3b) considering the approach as the experimental unit.

### Thoracoscopy procedure

All procedures were performed on standing calves, except in two cases when the animals had to be recumbent because of prior injury. Animals were immobilized with a head halter and a chute. Meloxicam (Metacam, Boehringer Ingelheim Vetmedica, Ingelheim am Rhein, Germany) was delivered intravenously (0.5 mg/kg), and lidocaine 2% and adrenaline 2‰ (in 5 ml; Xilocaína, Laboratorios Ovejero S.A., León, Spain) were administered subcutaneously to the puncture area 15 min before the first incision. Sedation was unnecessary, except in four cases (4 of 19, 20%) when the animals were very temperamental (1 ml of 2% Xylazine, delivered intravenously; Rompun, Bayer Iberia S.L., Barcelona, Spain). Sedation was administered on the right side of the animal.

**Fig. 3:**
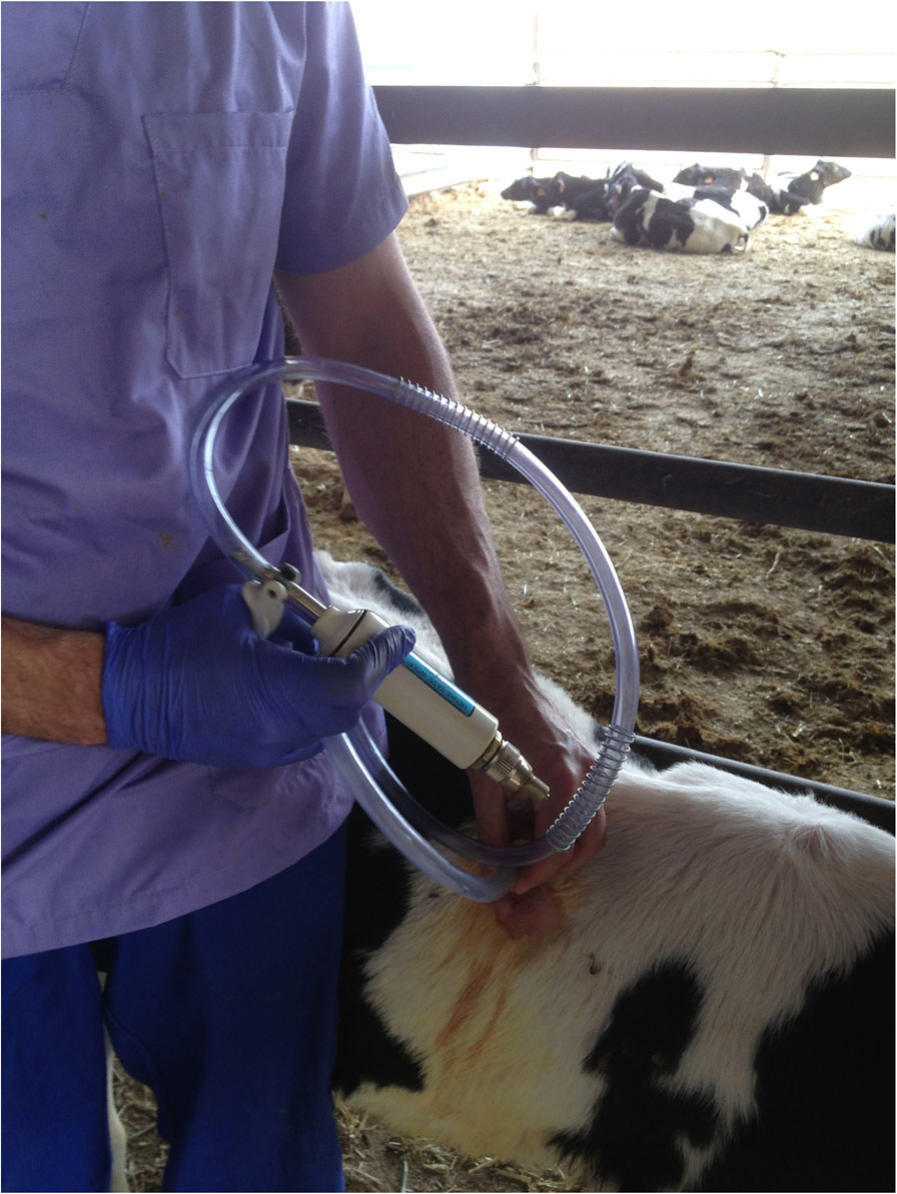
Handmade air evacuation system to reduce subclinical pneumothorax after thoracoscopy in calves chronically affected with BRD

The surgical field was prepared by surgical scrubbing and shaving, then an incision 1 cm long was made parallel to the dorsum. A metal trocar was immediately introduced into the thoracic cavity to a depth of 3 cm without damaging lung parenchyma. Blunt dissection of the thoracic wall was performed by piercing the trocar through the intercostal muscles while rotating the trocar at the same time. This helped ensure that, after trocar removal, the muscle fibers would close the incision upon returning to their original position. The end of the cannula that did not penetrate into the thoracic cavity was covered with the finger of a gloved hand in order to prevent airflow into the cavity. The endoscope was then introduced through the cannula rapidly, after removing the finger from the end of the trocar, and the right cavity was explored. Intrathoracic air was withdrawn, when necessary, by manual extraction via a syringe attached to the end of the cannula (Fig. 3). After exploration, the cannula was removed and the skin incision sutured. In all procedures, image quality was sufficient, such that pneumothorax did not need to be induced.

### Parameters and measurements

Studied parameters were evaluated by the same researcher in all cases. Parameters were classified into four categories (Table 1): “Animal”, “Technical”, “Safety” and “Diagnostic”. Lung and pleura lesions found in the BRD-lungs were classified as acute if the lung tissue area showed normal air volume surrounded by swollen tissue with a smooth surface and hyperemic color; or as chronic, if the lung area was pale and compressed, containing smaller air volume than normal [6, 36]. Severe adverse events, defined as any life-threatening event occurring during or soon after thoracoscopy, were recorded for up to 28 days after thoracoscopy by two veterinarians during daily visits.

**Table 1:** Description of variables evaluated during thoracoscopy of calves chronically affected with BRD

Parameter abreviation^a^		Description^b^	Score/Time of measurement	Definition of score	Further explanation
Animal	T (°C)	Rectal temperature at surgery			
	BW (kg)	Body weight	BW1	BW at surgery	
			BW2	BW 22 d after surgery	
	BMP	Breaths per minute	BMP1	BPM pre-surgery	
			BMP2	BPM during surgery	
			BMP3	BPM post-surgery	
	F (mg/dL)	Serum fibrinogen	F1	F at surgery	
			F2	F 6 d after surgery	
Technical	t (min)	Duration of surgery			
	EA (mL)	Volume of extracted air			
	VS (1–4)	Visualization scope	1	Excellent	Complete view of cranial lobes and between
			2	Optimal	Complete view of cranial lobes
			3	Valid	Peripheral view of cranial lobes
			4	Invalid	No view of cranial lobes
	IQD (1–3)	Image quality description^c^	1	Excellent	
			2	Moderate	
			3	Bad	
Safety	PS (1–4)	Pain Score	1	None	No sign of pain
			2	Slight	1–3 vocalizations
			3	Moderate	>3 vocalizations
			4	High	>3 vocalizations and kicking
	RT (1–4)	Recovery time	1	Immediate	eating, drinking and/or ruminating during the first 15 min after surgery
			2	Fast	= RT 1 by 15–30 min after surgery
			3	Medium	= RT 1 by 30–60 min after surgery
			4	Late	= RT 1 at >60 min after surgery
	RL (1–3)	Risk of lung perforation or laceration	1	Low	>1 cm between cannula and lung
			2	Medium	~1 cm between cannula and lung
			3	High	<1 cm between cannula and lung
	IOC	Intraoperative complications			Description
	RC (%)	Risk of threatening passive collapse			Yes or no
Diagnostic	OBRD-L	observed BRD lesions	Adhesions		Yes or no
			Edema		Yes or no
			Emphysema		Yes or no
			Acute lesions		Yes or no
			Chronic lesions		Yes or no
			Abscess		Yes or no
			Others		

^a^Parameters are shown with their abbreviated name and, in parentheses, the units or possible range of values

^b^Parameter abbreviations are explained in this column

^c^Image clarity, light output, focusing capability and pixelation of zoomed images

### Data processing and descriptive statistics

Data were analyzed descriptively using SPSS 22 (IBM, New York, USA). Data for continuous variables were reported as arithmetic mean and standard deviation (SD); data for categorical variables, as frequency percentages; and data for ordinal variables, as median (range). Normality was assessed using the Shapiro-Wilk test, which is designed for small samples. Inter-group differences were assessed for significance using the non-parametric Kruskal-Wallis test; if the difference was associated with *P* < 0.05, it was further assessed using the Mann-Whitney test with Bonferroni *post hoc* adjustment. Differences in percentages were assessed with Chi-square tests.

## Results

All thoracoscopies from both study phases were performed with minimal difficulty or adverse events. Results are summarized in Table 2.

**Table 2:** Experimentally determined parameters during thoracoscopy procedures in calves chronically affected with BRD

Parameter		Phase 1 – first procedure (*n* = 3)	Phase 1 – repeated procedure (*n* = 3)	Phase 2 (*n* = 18)
Animal	T (°C)	38.7 ± 0.25	38.9 ± 0.45	39.4 ± 0.53
	BW1/BW2 (kg)	BW1 70.4 ± 7.33	BW2 85.3 ± 13.02	BW1 157.8 ± 79.74
	BMP1	68 ± 4	70 ± 5	69 ± 9
	BMP2	92 ± 11	90 ± 7	84.0 ± 16
	BMP3	80 ± 4	70 ± 6	69 ± 8
	F1 or F2	F1= 933.3 ± 197.68	F2= 600.0 ± 282.84	–
Technical	t (min)	16.0 ± 4.86	14.33 ± 4.5	14.8 ± 3.84
	EA (ml)	1900.0 ± 216.02	583.3 ± 824.95	0
	VS (1–4)	3 (2–4)	3 (3–3)	1 (1–1)
Safety	PS (1–4)	3 (3–4)	3 (3–3)	2 (2–4)
	RT (1–4)	3 (3–3)	1 (1–1)	2 (1–4)
	RL (1–3)	2 (1–3)	2 (2–2)	1 (1–3)
	RC (%)	0%	0%	5.6% (1/18)

Parameter abbreviations and scoring are described in Table 1. Values are shown as average ± SD, percentage or median (min-max)

### Phase 1

The three diseased animals examined in this phase suffered no peri- or postoperative adverse events, other than transitory polypnea during the procedure and light subcutaneous emphysema. Risk of threatening passive collapse was judged to be low in all cases (Table 2). All calves increased in body weight during the month after surgery, showing an average daily gain of 536 ± 206.15 g. Thoracoscopy did not increase plasmatic fibrinogen levels.

The thoracoscopy procedure lasted 8–23 min, though the first thoracoscopies in the first two animals took longer because two approaches were used per surgery. The volume of extracted air ranged from 1600 mL to 2100 mL in the first intervention, and from 0 to 1750 mL in the second procedure. None of the approaches led to a visualization score of “excellent”, while the ventral approach led to a score of “optimal” (Table 3).

**Table 3:** Experimentally determined technical and safety parameters for comparing different approaches used with rigid endoscopes (equipment set-up 1) during thoracoscopy of calves chronically affected with BRD

Approach^a^	VS a *vs*. b *P* = 0.012	PS a *vs. c P* = 0.022﻿;﻿ b *vs*. c *P* = 0.01	RT *P* = 0.101	RL a *vs. b P* = 0.012
Ventral (*n* = 2)	2 (2–2)^a^	3 (3–3) ^a^	3 (3–3)	3 (3–3)^a^
Mid (*n* = 5)	3 (3–3)	3 (3–3)^b^	1 (1–3)	2 (2–2)
Dorsal (*n* = 1)	4^b^	4^c^	3	1^b^

Parameter abbreviations and scoring are described in Table 1. Values are shown as median (min-max). Numbers in the same column with different superscripts differ significantly, with the corresponding exact *P*-values indicated at the top of each column

^a^See approaches in Fig. 1

Animal discomfort was moderate in all cases except for one calf that experienced greater discomfort following the dorsal approach. Recovery time, defined as the time to eating, drinking and/or ruminating, was <1 h in all cases and <15 min after the second intervention. Risk of lung laceration was higher with the ventral approach and lower with the dorsal (Table 3).

**Fig. 4:**
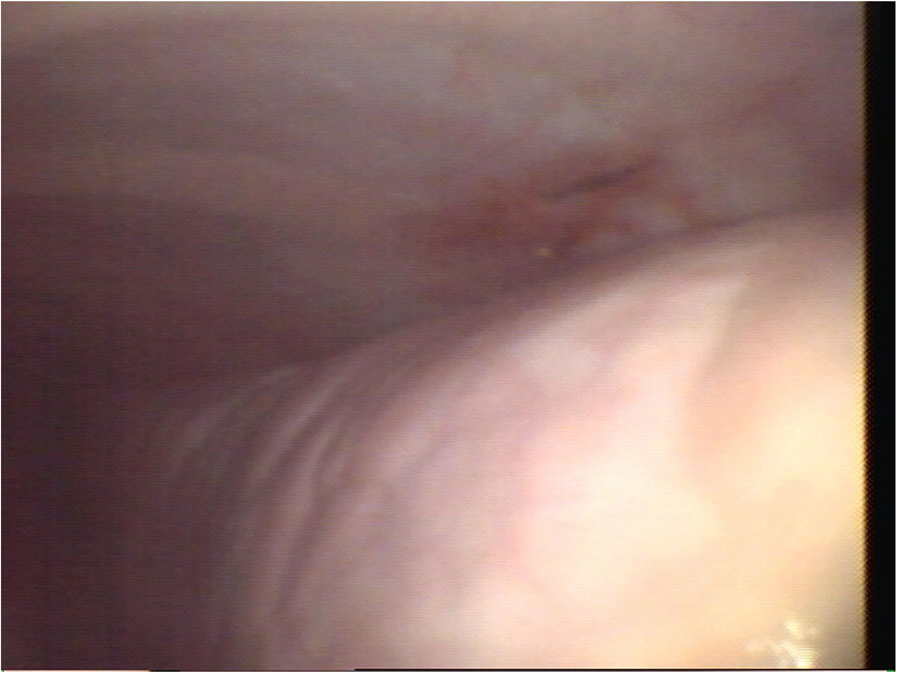
Thorascopic imaging and hyperemia in an area subjected to thoracoscopy 22 days before. The first and repeated thorascopies were performed using a rigid endoscope (equipment set-up 1). The calf was chronically affected with BRD

When the procedure was repeated on the same three animals 22 days later, no evidence of complications or lesions attributable to the first procedure was observed, except for a small hyperemic area at the incision site of the first thoracoscopy in one animal (Fig. 4) and small fibrin remains on the thoracic wall in another animal.

### Phase 2

The collected experience during the first phase of the study indicated that the dorsal approach allowed simpler manipulations to achieve lung tissue exploration, including easier lung perforation (Table 3), but that this approach was associated with greater discomfort due to the rigidity of the equipment. Therefore, all thoracoscopies in Phase 2 were performed using flexible optics introduced via the dorsal approach (Fig. 2), with the exception of two cases in which the procedure was performed on recumbent animals. Extraction of intrathoracic air was considered unnecessary based on previous experience.

The score for breaths per minute was moderate in all interventions, which lasted 14.78 ± 3.84 min. The visualization score was “excellent”. Flexible endoscopes proved to be superior to industrial videoscopes because of their higher image quality description (Table 4).

**Table 4:** Experimentally determined technical and safety parameters for comparing different equipment set-ups during thoracoscopy of calves chronically affected with BRD

Equipment set-up	VS^a^	IQD a *vs.* c *P =* 0.001; b *vs c P* = 0.005	PS a *vs.* b *P* < 0,0001	RT *P* = 0.043	RL a *vs.* b *P =* 0.006
1 (*n* = 8)	3 (2–4)	1 (1–1)^a^	3 (3–4)^a^	3 (1–3)	2 (1–3)^a^
2 (*n* = 5)	1 (1–1)	1 (1–1)^b^	2 (2–4)	3 (2–4)	1 (1–3)
3a (*n* = 9)	1 (1–1)	3 (3–3)	2 (2–2)^b^	2 (1–4)	1 (1–1)^b^
3b (*n* = 4)	1 (1–1)	2 (2–3)^c^	2 (2–2)	2.5 (1–4)	1 (1–1)

Parameter abbreviations and scoring are described in Table 1. Values are shown as median (min-max). Numbers in the same column with different superscripts differ significantly, with the corresponding exact *P*-values indicated at the top of each column

^a^Inter-group differences were not assessed for statistical significance, since VS depends exclusively on approach and is independent of equipment type

The Pain score was “slight” in 15 procedures. In 12 procedures, recovery time was <30 min. In one case, a light pleural laceration occurred in one calf because it was lying down during the thoracoscopy due to an old femur fracture. This calf was euthanized the day after thoracoscopy with the farmer’s consent. Another calf was euthanized 7 days after thoracoscopy when persistently BVD infection was confirmed. Risk of threatening passive collapse was judged to be low in all cases.

**Fig. 5:**
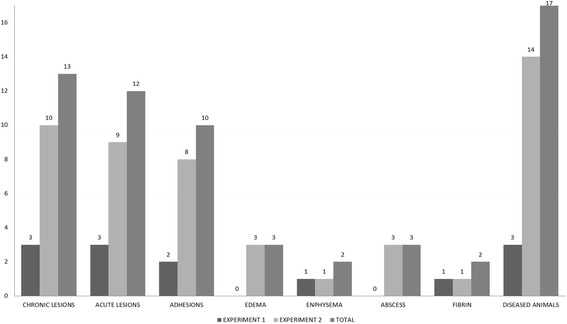
Thoracoscopic imaging of BRD lesions using different equipment set-ups in calves chronically affected with BRD. Values refer to numbers of animals

**Fig. 6:**
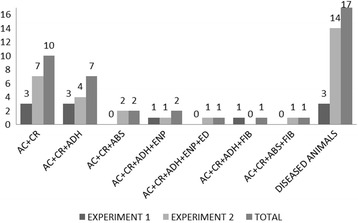
BRD lesions observed by thoracoscopy performed using different equipment set-ups. All images are of calves chronically affected with BRD. AC, acute lesion; CR, chronic lesion; ADH, adherence; ABS, abscess; ENP, emphysema; ED, edema; FIB, fibrin. Values refer to numbers of diseased animals

**Fig. 7:**
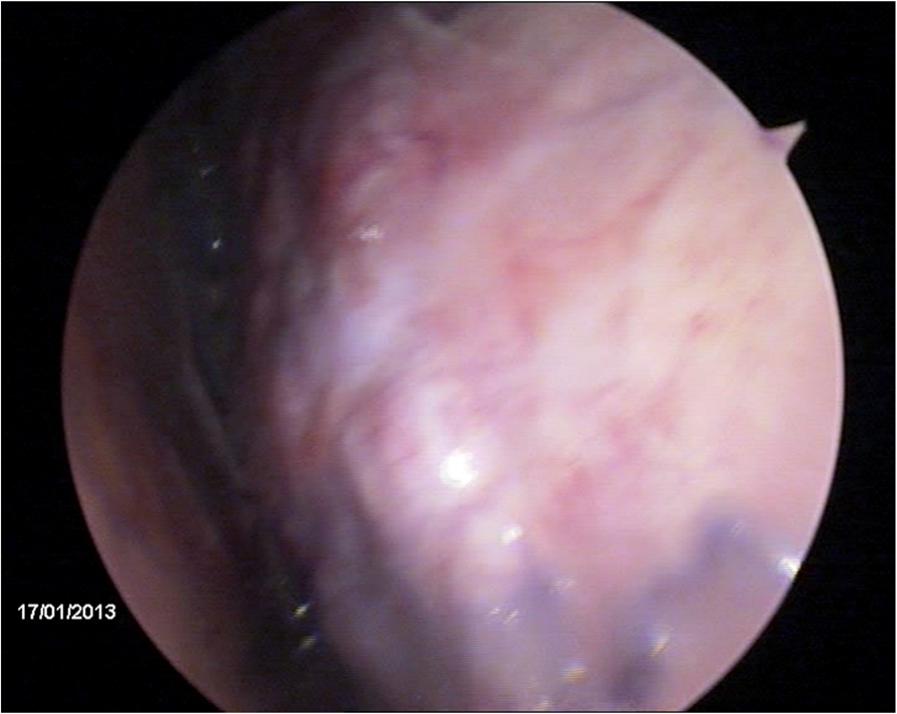
Lung abscesses observed by thoracoscopy performed using a flexible endoscope (equipment set-up 2) in a calf chronically affected with BRD

Since the study was not designed to prove the diagnostic power of the technique, only selected calves with a medical history of chronic BRD and two healthy calves were used. Different lung lesions were found in all BRD-affected calves, while no pneumonic lesions were observed in healthy animals (Figs. 2, 5 and 6). Three animals presented small lung abscesses (Fig. 7) with patterns consistent with *Mycoplasma bovis* infection.

### Comparison of equipment set-ups

Set-up 1 (rigid) allowed more complete observation of cranial lobes when the endoscope was introduced via the ventral approach than via the dorsal approach, even though the dorsal approach was safer (*P* < 0.05, Table 3). The ventral approach was also associated with a lower pain score than the dorsal approach (3 vs. 4; *P* < 0.05, Table 3). On the other hand, the ventral approach did not allow complete visualization of the caudal lobe, and it was associated with high perforation risk (Table 3). In contrast, flexible optics (set-ups 2, 3a and 3b) introduced via the dorsal approach could enter safely between the cranial lobes, allowing close and peripheral visualization of lung tissue without compromising animal welfare. In fact, all intrathoracic structures could be examined after only one insertion of the optics, and flexible optics were more comfortable than rigid optics for most calves (*P* < 0.05, Table 4).

Set-up 1 provided images with an excellent image quality score (Table 2), making it superior to set-ups 3a and 3b, which involved industrial videoscopes with, respectively, low- and high-quality imaging capability. Nevertheless, the images obtained with set-up 1 were similar in quality to those obtained with flexible endoscopes (Table 4).

## Discussion

The results of the present study support thoracoscopy as an amenable and safe potential diagnostic technique for BRD-affected calves under field conditions. Using flexible endoscopes (equipment set-up 2) inserted via the dorsal approach (Fig. 2) with standing animals, we were able to explore the right lung in approximately 15 min without compromising calf health or welfare. We were able to detect lesions in all of the 17 diseased calves in our study.

While clinical criteria alone may lead occasionally to misdiagnosis of chronic BRD, we were careful in our study to include only animals that had presented classic BRD symptoms demonstrated to be high specifically associated with BRD disease [35], associated to a delay in growth and weight in a continuous way for at least one month prior to our work [34].

While thoracoscopy has been used for decades in horses [37], studies of endoscopes in cows have appeared only recently [25, 32], and these involved rigid set-ups and standing healthy cows. The present study is, to our knowledge, the first to examine thoracoscopy in several diseased animals and to compare rigid and flexible equipment set-ups as well as different approaches under field conditions. We also systematically examined possible effects of the procedures on animal welfare and safety.

All calves increased in body weight during the month after surgery, showing an average daily gain of 536 ± 206.15 g. This is lower than the average daily gain of 1200 g reported for healthy feedlot mates living under similar conditions [38].

We found that the best combination was flexible equipment and the dorsal approach. Higher pain scores were observed when rigid equipment was used with the dorsal approach, although this procedure gave the highest visualization score. Higher pain scores were also observed when flexible equipment was used with the ventral approach.

Flexible endoscopes (equipment set-up 2) showed obvious advantages, as demonstrated in humans [39, 40]. With a flexible set-up, we were able to visualize the entire thoracic cavity relatively quickly with a single penetration, in contrast to the experiences reported when using rigid equipment for adult cows [25]. Image quality with the industrial videoscopes that we tested was not optimal. Nevertheless, their relatively low cost may make them attractive, and the real-time images may be adequate to detect BRD in advanced phases. However, they do not allow image recording, nor do they have the capability to take biopsies or remove adhesions.

An approach from the right-hand side was selected because necropsy studies indicate that right lung examination allows primary pulmonary disease diagnosis [29, 41–43]. Since most animals with BRD present with bilateral lung lesions [44], we assumed that the right-hand side was representative of the overall lesion pattern. On the other hand, exploring only the right lung can miss up to 16% of lesions [42] or even 30% [16]. Further studies should investigate the optimal procedure for approaching the left lung when necessary. Regardless of which lung is explored, the cranial lobes should be examined completely, since they are the first areas to be affected in BRD and are among the worst-affected areas [42, 45].

All thoracoscopies were completed within 23 min, which is comparable to reports in cows (30 min [25], 20 min [32]) and horses (15 min [41]). Those previous studies were performed under controlled conditions in a hospital, whereas our study was performed under field conditions. Sedation may be necessary to ensure the safety of animals and operators when the animal is temperamental, which was the case with 4 of 19 animals in our study.

The adverse events most often associated with thoracoscopy include hemorrhage and trauma to adjacent structures within the thoracic cavity [46], mild subcutaneous emphysema and subclinical pneumothorax [41]. In our study, the only complication observed was minor transient subcutaneous emphysema. Laceration occurred in only one calf, which had to be recumbent during the procedure. This recumbent position impeded visualization of the cranial area, and the procedure was interrupted. We conclude, therefore, that thoracoscopy should be performed in standing animals, as described for horses [29, 41].

No calf in our study showed lung collapse. While passively introduced air was extracted in Phase 1 of the study, such extraction was deemed unnecessary in the 18 procedures of Phase 2. We did not need to infuse CO_2_ to improve visualization, in contrast to a previous study in cows [32], nor did we need to administer oxygen intranasally, in contrast to the report of a pericardiotomy in a cow [33]. Postsurgical antibiotherapy was unnecessary during our study, even though most of our procedures were performed under field conditions. In fact, no infectious complications were observed 28 days after surgery. No mortality was observed during the same period, except for two animals that were euthanized for humanitarian reasons. Another study also reported low mortality and complication rates after thoracoscopy in small animals [46].

We did not include post-mortem examination because we did not want to impact the productivity of the animals involved, which was necessary to gain the support of the farm owners involved. Therefore, the animals in our study were later fattened and slaughtered as per normal farm procedure. Future studies should verify our findings using post-mortem examination.

**Fig. 8:**
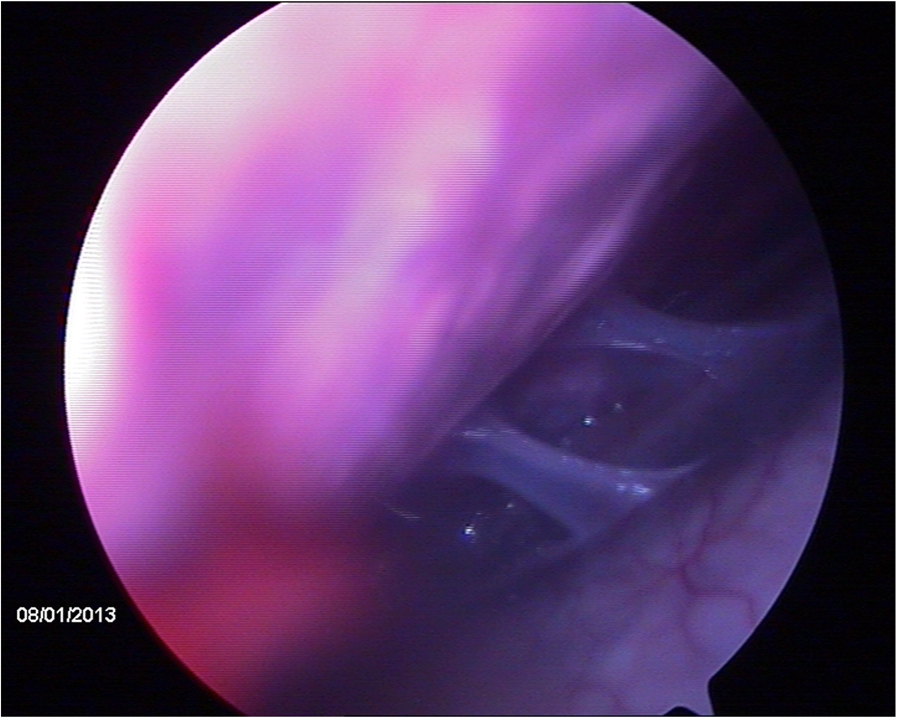
Lung adhesions observed by thoracoscopy performed using a flexible endoscope (equipment set-up 2) in a calf chronically affected with BRD

Although the present study was not designed to demonstrate diagnostic capability, our results show that thoracoscopy was able to detect several lesions (Figs. 5, 6, 7 and 8), supporting the potential usefulness of thoracoscopy as a complement to established techniques for diagnosing BRD. Thoracoscopy may be particularly useful because it allows sequential biopsying of live animals. In humans, thoracoscopy has been shown to enhance the sensitivity and specificity of lung biopsy-based diagnosis [47]. Thoracoscopy may facilitate studies based on sequential-biopsy lung mapping in order to clarify the disease process, support preclinical research and clinical trials, and perform pharmacovigilance. Therefore, this study establishes the feasibility and potential suitability of thoracoscopy for diagnosing calves infected with BRD. This justifies larger studies aimed at rigorously assessing the technique’s sensitivity and specificity.”

## Conclusions

Thoracoscopy has been demonstrated for the first time to be an easy, safe and rapid exploratory technique that could be applied under field conditions to animals affected by BRD.
